# Cohort differences between preschool development of in vitro fertilization and naturally conceived infants

**DOI:** 10.1097/MD.0000000000038190

**Published:** 2024-07-05

**Authors:** Chunyan Guo, Jingcai Wang, Lixin Yang, Yanqiu Wu, Xia Liu, Qili Zhou

**Affiliations:** a Department of Neonatology, Affiliated Hospital of Chengde Medical College, Chengde, Hebei, China.

**Keywords:** assisted reproductive technology, in vitro fertilization, naturally conceived children, preschool age, psychomotor development

## Abstract

To explore the differential cohort situation between preschool development of in vitro fertilization (IVF) and naturally conceived infants. From April 2014 to June 2022, 60 preschool IVFs were selected as the research subjects for follow-up at the pediatric health clinic of hospital’s prevention and health department. They were set as the experimental group (Group S), and 60 naturally conceived infants of the same age were selected as the control group (Group Z). Data from both groups were collected through telephone follow-up and other methods. No significant difference showed between the 2 groups in age specific height, age specific weight, Gesell developmental score, Denver developmental screening test screening results, intellectual development index, and motor development index (*P* > .05). The influence of birth environment factors such as family background and maternal education level on children’s height and weight was not significant (*P* > .05), while maternal education level had a significant impact on children’s intellectual development index (*P* < .05). No significant difference showed in the development of preschool children in IVF compared to naturally conceived children, and the level of parental education has a significant impact on children’s mental and motor development.

## 1. Introduction

With the aggravation of environmental pollution, the pressure of work and life, and the postponement of childbearing age, the incidence rate of infertility is increasing. In China, the incidence rate of infertility is 7% to 10%, leading to a rising demand for pregnancy. In this context, assisted reproductive technology (ART) has been continuously innovated and optimized, and applied in clinical practice to help some infertile patients conceive children.^[1–3,31]^ According to the treatment needs of different populations, different ART technologies have been developed, such as in vitro fertilization (IVF), embryo transfer, and blastocyst culture. The application of assisted reproductive technologies such as IVF and embryo transfer in clinical practice has greatly improved the live birth rate and clinical pregnancy rate.^[27,28]^ The rate of IVF births has significantly increased, accounting for approximately 1% to 3% of all newborns. With the help of ART technology, many patients have realized their dreams of giving birth. At the same time, due to differences from natural conception, these parents may worry about whether ART technology will have an impact on their children’s development and other aspects, doubt the safety of the technology, and even panic about the safety of mother and baby.^[4–6]^ Therefore, the pregnancy and offspring health of IVF are receiving increasing attention from patients and families, and its safety issues have become a research hotspot. Natural pregnancy is a product of natural selection, and assisted pregnancy requires a series of precise and strict in vitro procedures. Some scholars believe that these invasive and nonphysiological methods avoid natural conception, which may lead to abnormal fertilization of sperm and eggs, resulting in a decrease in embryo quality, and even the transmission of pathogenic genes to the next generation, thereby affecting the health of the next generation. Some scholars have studied the offspring of assisted pregnancy and compared them with those of natural pregnancy. They have found that compared to the offspring of natural pregnancy, the probability of premature birth in IVF is higher, and this type of baby has a relatively lighter weight after birth. In order to understand the changes in the growth and development process of IVF and naturally conceived infants, this study focuses on these 2 types of children and analyzes whether there are differences in their growth and development in the preschool stage, in order to provide valuable reference data for IVF in this growth stage.

## 2. Materials and methods

### 2.1. Research object

From April 2014 to June 2022, a simple random sampling method was used to select IVF. Sixty preschool IVFs were followed up at the pediatric health clinic of a certain hospital’s preventive health department as the research subjects, and set as the experimental group (Group S). During the same time period, 60 naturally conceived infants of the same age group were selected as the control group (Group Z). The relevant research plan was approved by the hospital ethics committee. In terms of children’s families, they had sufficient understanding of the research and signed informed consent forms.

Inclusion criteria for research subjects: The selected age range is between 3 and 6 years old; Children are all born in cities; No history of otitis media or other medical conditions in the past; Children have good compliance; No functional impairment of limbs; No mental disorders; Parents have no family history of hereditary diseases; Parents do not have chromosomal diseases. Exclusion criteria: The age of IVF does not meet the inclusion criteria; Children have severe visual and auditory impairments; Children have limb dysfunction; Children suffer from congenital heart disease and other diseases; Incomplete information on children and their families; The intellectual development level of children is severely lagging behind; Parents have a family history of hereditary diseases; Parents do not have chromosomal diseases; Children’s language is not Chinese; Children’s cooperation is not high and cannot meet the requirements of the experiment; Unable to cooperate with follow-up; Children may experience suffocation at birth.

### 2.2. Data collection methods

The relevant information of preschool children was collected by means of telephone follow-up, original data query, and face-to-face inquiry. The children’s birth gestational weeks, birth weight, and whether there was asphyxia were understood. The family background of the children was recorded, including the mother’s age at childbirth, parents’ educational background, average annual household income, parents’ marital status, and family origin. The mothers’ prenatal conditions were inquired about to understand whether there were any pregnancy complications. Queries were conducted regarding the parents’ genetic factors and reproductive issues. Additionally, the occurrence of diseases requiring hospitalization for more than 7 days in the neonatal, infant, and preschool stages was also recorded for 2 groups of children. Under the category of living environment factors, it can be divided into 5 aspects: household income level, mother’s and father’s education levels, family origin, and marital status. In terms of household income level, it can be classified into low, medium, and high 3 levels, with low-level households (<10,000 yuan annual income), medium-level households (10,000–50,000 yuan annual income), and high-level households (>50,000 yuan annual income). In the classification of education level, it can be divided into 3 levels: primary, secondary, and tertiary. Primary education level corresponds to education level below high school, secondary education level corresponds to high school and technical secondary school, and tertiary education level corresponds to college and above. In terms of family origin, it is divided into urban, town, and rural areas. Regarding marital status, it also includes 3 situations: first marriage, remarriage, and divorce. When investigating parental reproductive issues, factors of male and female infertility were understood. In the investigation of parents’ genetic factors, it was inquired whether both parents had hereditary diseases or chromosomal abnormalities.

### 2.3. Evaluation method

A group of professionally trained physicians was selected to conduct physical examinations on 2 groups of preschool children, assessing their height and weight. The growth and development of the preschool children were evaluated using the World Health Organization (WHO) recommended reference values for height and weight for children aged 0 to 6 years, through their physical development assessment forms. The children’s height and weight were categorized into 3 levels: below average, average, and above-average. By considering the weight-for-age, the developmental status of the preschool children could be understood, reflecting whether there was delayed development or short-term and long-term nutritional status. This indicator was not only applicable for individual evaluation but also for evaluating groups, showing sensitivity in observing short-term changes. Height-for-age could be used to detect long-term nutritional status and could be influenced by certain genetic factors. By considering both height-for-age and weight-for-age, the nutritional status in both the short-term and long-term could be comprehensively reflected. The wechsler preschool and primary scale of intelligence (WPPSI) were used to evaluate the intellectual development of the preschool children, consisting of 2 main parts: verbal IQ and performance IQ, each part further divided into 5 aspects. The verbal IQ section assessed vocabulary, general knowledge, and other related content, while the performance IQ section included geometric patterns, puzzles, and other tasks, with a total of 54 items on the scale. During the evaluation process, a quiet environment was selected to conduct one-on-one tests on the preschool children, ensuring that the children were in a good state, alert, and without hunger. The Chinese Child Development Scale (3–6 yrs) was utilized to evaluate the psychomotor development of the preschool children, employing a blind method during the assessment. The scale consisted of 2 subtests: intelligence and motor skills. The intelligence subtest included items such as quantifier usage, language comprehension, and finding pictures according to examples, totaling 11 items. The motor skills subtest included tasks such as single-leg standing, standing long jump, and fast picking of small beans, with a total of 5 items. Through the previous scale, the Mental Development Index (MDI) reflecting intellectual development was obtained, while the latter scale provided the Psychomotor Development Index (PDI) indicating motor development.^[10–12]^ Both indices consisted of 7 levels, ranging from average to above-average, with higher levels corresponding to higher scores, indicating better psychomotor development in children. The Gesell Developmental Diagnostic Scale was selected, consisting of 5 aspects: language, gross motor skills, fine motor skills, adaptive behavior, and personal-social behavior. This scale assessed children’s neurological and psychological development, understanding the developmental age and quotient of each aspect. A higher developmental quotient indicated better overall development in children. Trace elements in the children’s venous blood were tested, requiring a 4-hour fasting period prior to the test. After the test, the zinc and iron content in the children’s venous blood was determined. Hemoglobin (Hb) content in the children’s blood was also measured, with blood samples collected from the left ring finger. A spectrophotometer was selected to perform the relevant assays. When the hemoglobin level was below 110 g/L, anemia in the children was diagnosed. The Denver Developmental Screening Test (DDST), consisting of 104 items, was chosen to conduct developmental screening on the children, yielding 4 possible results: normal, suspicious, delayed, and unexplainable. This test was employed to assess the children’s intellectual abilities and examine their cognitive status.^[13,14]^

### 2.4. Statistical methods

SPSS 23.0 was selected to organize and analyze the relevant data of 2 groups of preschool children and their parents. The collected data consisted of 2 types: quantitative data and categorical data. The former type of data was analyzed using *t*-tests, and its format was presented as mean ± standard deviation. The latter type of data was represented as a percentage (%), and correlation analyses were conducted using *χ*^2^ tests. During the comparison process, a significance level of *P* < .05 indicated significant differences with statistical significance.

## 3. Results

### 3.1. Basic data analysis of Z and S groups

The relevant basic information of 2 groups of preschool children and their parents was organized, with both groups consisting of 60 cases. When comparing the basic information of the 2 groups, there was no statistically significant difference (*P* > .05) in family factors such as family background, maternal education level of university or above, annual family income ≥ 50000 yuan, and maternal age ≥ 35 years. However, a significant difference (*P* < .05) showed in marital status and paternal education level of university or above. In terms of the children’s situation, no significant difference (*P* > .05) showed between the 2 groups premature birth (<37 wk), gender, low birth weight (≤2.5 kg), and previous hospitalization history. However, a significant difference showed (*P* < .001) in the cesarean section indicator. The basic information of the preschool children and their parents in groups Z and S is shown below (Table 1).

**Table 1:** Basic information of 2 groups.

Characteristic	Group Z (n = 60)	Group S (n = 60)	*χ* ^2^	*P*
Family factors (Cases, %)	/	/	/	/
Family origin (city)	57 (95.00%)	60 (100.00%)	1.367	.242
Marital status (first marriage)	53 (88.33%)	60 (100.00%)	5.461	.019
Mother’s college and above education	31 (51.67%)	22 (36.67%)	0.0001	.993
Father’s college or above education	33 (55.00%)	24 (40.00%)	6.380	.012
Family annual income ≥ 50000 yuan	25 (41.67%)	20 (33.33%)	0.569	.451
Mother’s age ≥ 35 years old	3 (5.00%)	6 (10.00%)	0.481	.488
Children’s situation (Cases, %)	/	/	/	/
Premature delivery (<37 wk)	5 (8.33%)	6 (10.00%)	0.000	1.000
Low body weight (≤ 2.5 kg)	3 (5.00%)	0 (0.00%)	1.368	.242
Gender (male)	26 (43.33%)	35 (58.33%)	2.134	.144
Cesarean section	25 (41.67%)	60 (100.00%)	46.629	<0.001
Previous hospitalization history	14 (23.33%)	12 (20.00%)	0.049	.825

### 3.2. Height and weight of children

Table 2 shows the height and weight of children in groups Z and S. In terms of age-specific height and weight, the levels were different, and the corresponding number of children in the 2 groups was also different. Among them, in terms of age-specific height, there were 38 children in group S at the medium-level, which was 2 more than the 36 children in group Z. 18 children were in the medium or above level, which was 1 less than that of group Z. In terms of age-specific weight, the number was the same in both groups, with both being 42. The number of children in the medium or above level was 18 in group S, which was 4 more than that in group Z. The number of children in the medium or below level was 0 in group S, which was 4 less than that in group Z. Overall, compared with group Z, group S had slightly more preschool children at the medium and medium or above level, but the differences in age-specific height and weight were not significant (*P* > .05).

**Table 2:** Height and weight of children.

Characteristic	Group Z (n = 60)	Group S (n = 60)	*χ* ^2^	*P*
Age specific height (Cases, %)	/	/	/	/
Under-intermediate-level	5 (8.33%)	4 (6.67%)	0.000	1.000
Medium	36 (60.00%)	38 (63.33%)	0.035	.851
Medium or above	19 (31.67%)	18 (30.00%)	0.000	1.000
Age specific weight (Cases, %)	/	/	/	/
Under-intermediate-level	4 (6.67%)	0 (0.00%)	2.328	.127
Medium	42 (70.00%)	42 (70.00%)	0.040	.842
Medium or above	14 (23.33%)	18 (30.00%)	0.032	.858

### 3.3. Evaluation of Hb content and detection of trace element content in groups Z and S

Table 3 presents the evaluation of Hb content and detection of trace element content in children in groups Z and S. In terms of Hb content, there were slightly more normal preschool children in group Z than in group S, with corresponding numbers of 56 and 55, respectively. The number of children with abnormal Hb content was relatively small in both groups, with both being <10.00%. Among them, the number of children with abnormal Hb content in group S accounted for 8.33% of all children. In terms of trace element deficiencies, there were relatively more children with deficiencies in group Z, especially those with iron deficiency, with 10 cases, which was 4 more than that in group S, which had 6 cases. Group S had 2 children with calcium deficiency, while group Z had 3. There was only 1 child with magnesium deficiency in group S, which was 1 less than the 2 in group Z. Overall, the differences in Hb content evaluation and trace element content were not statistically significant (*P* > .05).

**Table 3:** Evaluation of Hb content and detection of trace element content.

Characteristic	Group Z (n = 60)	Group S (n = 60)	*χ* ^2^	*P*
Evaluation of Hb content (Cases [%])	/	/	/	/
normal	56 (93.33%)	55 (91.67%)	0.120	.729
Abnormal	4 (6.67%)	5 (8.33%)		
Lack of trace elements (Cases, %)	/	/	/	/
Magnesium deficiency	2 (3.33%)	1 (1.67%)	0.342	.559
Copper deficiency	1 (1.67%)	2 (3.33%)	0.342	.559
Calcium deficiency	3 (5.00%)	2 (3.33%)	0.209	.648
Zinc deficiency	2 (3.33%)	1 (1.67%)	0.342	.559
Iron deficiency	10 (16.67%)	6 (10.00%)	0.649	.420

### 3.4. Disease incidence in children in groups Z and S

**Figure 1.**
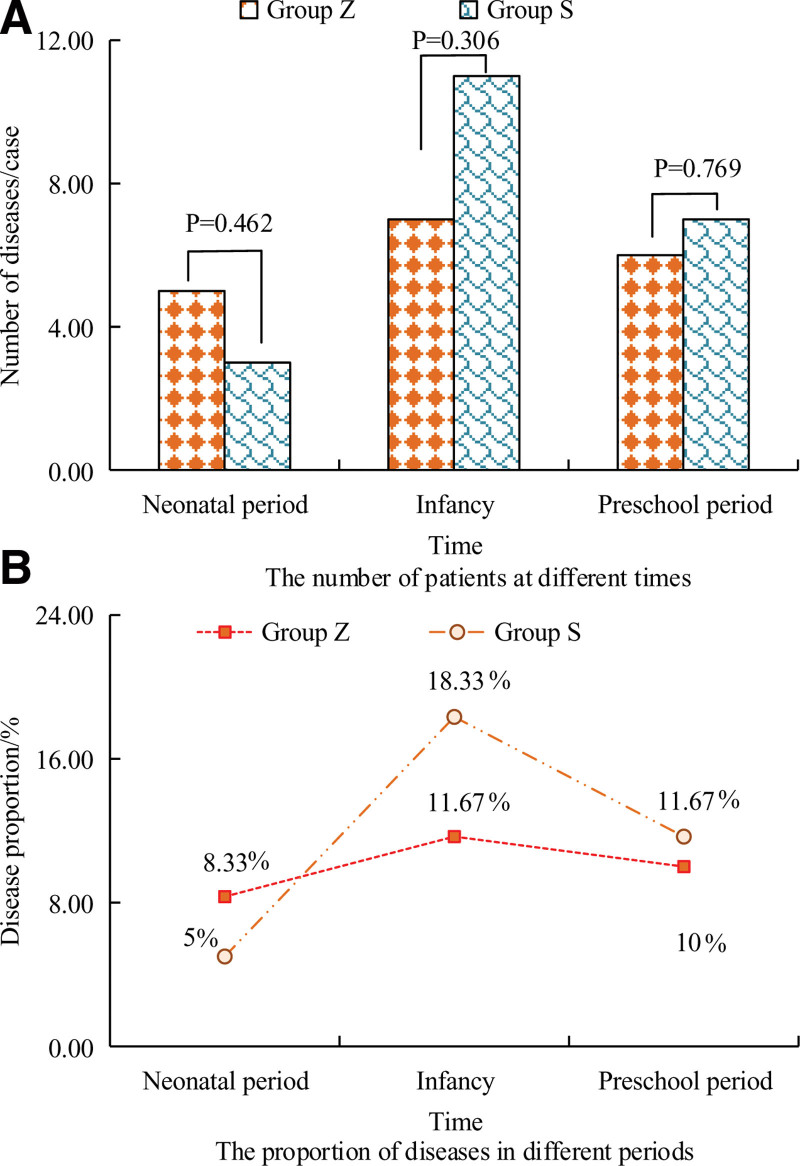
Disease incidence in children in groups Z and S.

The situation of disease occurrence in different stages of children in groups Z and S is depicted in Figure 1. Differences were observed in the number of disease occurrences among children in groups Z and S at different stages. In the newborn period, the children in group Z was slightly more, while in the infant and preschool periods, the children in group Z was slightly fewer. Specifically, in the newborn period, the number of children with disease occurrences in group Z and group S was 5 and 3 cases, respectively, indicating an excess of 2 cases in the former group compared to the latter. During the preschool period, there were 7 cases of disease occurrence among children in group S, accounting for 11.67% of all children in that group. At this time, the probability of disease occurrence among children in group Z was 10.00%, corresponding to 6 cases. Overall, although the number of disease occurrences was slightly higher in group S, no significant difference showed between the 2 groups (*P* > .05).

### 3.5. Gesell developmental score and wechsler preschool and primary scale of intelligence score in groups Z and S

**Figure 2.**
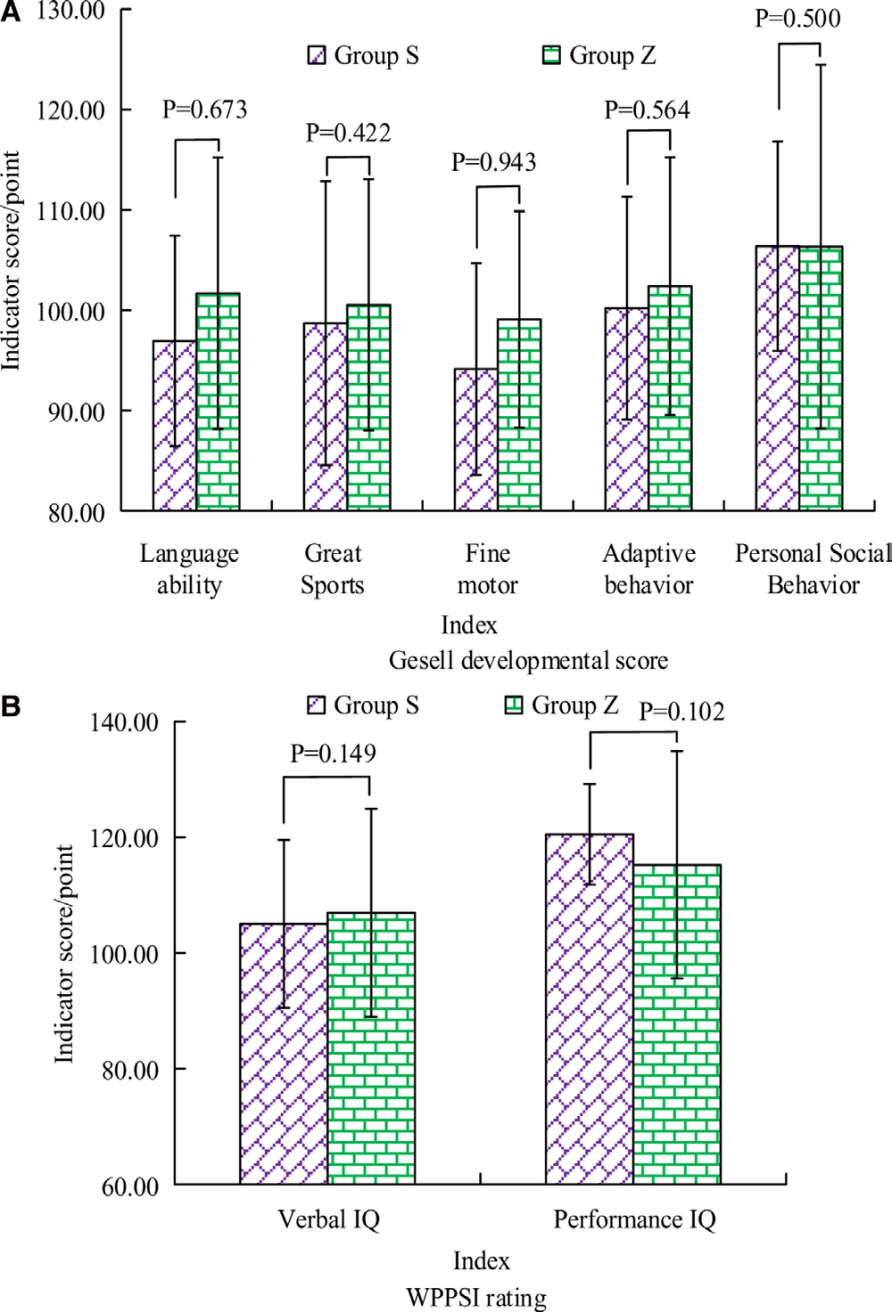
Gesell developmental score and WPPSI score of children in groups Z and S. WPPSI = wechsler preschool and primary scale of intelligence.

Figure 2 displays the Gesell developmental scores and WPPSI scores of children in groups Z and S. In terms of Gesell developmental scores, the scores for language ability, gross motor skills, fine motor skills, and adaptive behavior were slightly lower in group S, while the scores for personal-social behavior were slightly higher in group S. Although there were slight differences in the Gesell developmental scores between children in group Z and group S (*P* > .05). Regarding the WPPSI scores, the language IQ score was slightly lower in group S, whereas the performance IQ score was slightly higher in group S. Overall, no significant differences showed in the WPPSI scores between the 2 groups of children (*P* > .05).

### 3.6. Analysis of intellectual development of children in group Z and S

The intellectual development of children in groups Z and S is presented in Table 4. In Table 4, there were certain differences observed in the scores corresponding to different dimensions between the 2 groups of children. In the dimensions of geometric shapes, puzzles, analogies, vocabulary, and general knowledge, the scores corresponding to group Z were slightly lower than those of group S. Conversely, in the dimensions of animal rooms, picture completion, block patterns, and arithmetic problems, the scores corresponding to group Z were slightly higher than those of group S. Overall, no significant differences showed in the scores related to intellectual development between children in 2 groups (*P* > .05).

**Table 4:** Intelligence testing of children in groups Z and S.

Dimension (Points)	Group Z (n = 60)	Group S (n = 60)	*t*	*P*
Geometry	14.79 ± 2.91	14.98 ± 2.86	0.361	.719
Animal room	13.48 ± 4.13	12.90 ± 3.45	0.835	.406
Labyrinthine fluid	13.30 ± 2.59	13.66 ± 2.67	0.750	.455
Drawing supplementation	10.67 ± 2.37	10.21 ± 2.46	1.043	.299
Wooden block pattern	12.89 ± 2.34	12.24 ± 2.58	1.446	.151
Analogous words	10.62 ± 2.87	11.12 ± 2.52	1.014	.313
Vocabulary	8.59 ± 2.53	9.23 ± 2.67	1.348	.180
Common sense	11.98 ± 0.78	12.10 ± 0.45	1.032	.304
Problem in arithmetic	16.25 ± 2.45	15.49 ± 2.78	1.589	.115

### 3.7. Denver developmental screening test screening status of children in groups Z and S

Table 5 presents the DDST screening results for children in groups Z and S. Variances were observed in the DDST screening results for children of different ages in groups Z and S. The number of children with suspected developmental delay identified in the DDST screening was slightly lower in group Z for 3-year-old and 5-year-old children, while it was slightly higher in group Z for 4-year-old children. The number of children with suspected developmental delay identified in the DDST screening was the same for 6-year-old children in both groups. Specifically, there were 2 cases of suspected developmental delay identified in the DDST screening for 3-year-old children in group Z, which was 1 case less than in group S, where there were 3 cases. For 5-year-old children, the number of cases with suspected developmental delay identified in the DDST screening was 4 and 5 for groups Z and S, respectively, corresponding to proportions of 6.67% and 8.33%. Overall, no significant differences showed in the numbers of children with suspected developmental delay identified in the DDST screening (*P* > .05).

**Table 5:** DDST screening for children in groups Z and S.

Suspicious situations of different ages		Group Z (n = 60)	Group S (n = 60)	*χ* ^2^	*P*
3-year-old	Number of cases	2	3	0.209	.648
	Probability (%)	3.33	5.00		
4-year-old	Number of cases	4	2	0.702	.402
	Probability (%)	6.67	3.33		
5-year-old	Number of cases	4	5	0.120	.729
	Probability (%)	6.67	8.33		
6-year-old	Number of cases	4	4	0.000	1.000
	Probability (%)	6.67	6.67		

### 3.8. The psychomotor development of children in groups Z and S

Table 6 presents the psychomotor development status of children in groups Z and S. Compared to group Z, children in group S had slightly higher scores in PDI and MDI, but there were no significant differences in scores for these 2 indicators (*P* > .05). However, in terms of PDI and MDI scores ≤ 69, the occurrence rate was lower in group S (*P* < .05). Specifically, the occurrence rate of PDI scores ≤ 69 in children from group S was 1.67%, which was 11.66% lower than that of group Z, which was 13.33%.

**Table 6:** The psychomotor development of children in groups Z and S.

Index		Group Z (n = 60)	Group S (n = 60)	*T (χ*^2^)	*P*
PDI (points)		87.79 ± 13.25	89.45 ± 10.37	0.764	.446
MDI (points)		87.46 ± 15.49	89.71 ± 13.22	0.856	.394
PDI < 80	Number of cases	15	9	1.875	.171
	Probability (%)	25.00	15.00		
MDI < 80	Number of cases	23	24	0.035	.852
	Probability (%)	38.33	40.00		
PDI ≤ 69	Number of cases	8	1	5.886	.015
	Probability (%)	13.33	1.67		
MDI ≤ 69	Number of cases	7	1	4.821	.028
	Probability (%)	11.67	1.67		

### 3.9. The impact of birth environment on children’s mental development index and psychomotor development index

Table 7 presents the impact of birth environment on the MDI and PDI of 120 children. Multiple linear regression analysis was conducted with independent variables such as family background and other influencing factors, and dependent variables including PDI and MDI. After the analysis, it was found that group, family background, age, marital status, and family annual income had no statistically significant impact on PDI and MDI (*P* > .05). However, the father’s education level had an impact on PDI, with statistical significance (*P* < .05), and the mother’s education level had a significant impact on MDI (*P* < .05).

**Table 7:** The impact of birth environment on children’s MDI and PDI.

Influence factor	PDI		MDI	
	*t*	*P*	*t*	*P*
Group	0.921	.359	0.978	.328
Family origin	0.113	.912	1.919	.057
Mother’s education level	–0.534	.589	2.382	.016
Father’s educational level	2.451	.012	–1.581	.117
Age	0.302	.758	–1.798	.074
Marital status	1.719	.090	–0.182	.858
Annual household income	–1.181	.242	0.618	.535

### 3.10. The influence of birth environment on children’s height and weight

Table 8 presents the impact of birth environment on the age-specific height and weight of 120 children. Logistic regression analysis was employed in the multifactor analysis. Upon analysis, it was found that factors such as group, family background, mother’s level of education, father’s level of education, age, marital status, and family annual income had minimal impact on the height and weight of the children, and there was no statistical significance (*P* > .05).

**Table 8:** The influence of birth environment on children’s age specific height and weight.

Influence factor	Weight-for-age			Height-for-age		
	Wald value	b value	*P*	Wald value	b value	*P*
Group	2.550	1.243	.109	1.138	0.809	.287
Family origin	0.003	–0.041	.951	0.031	–0.101	.859
Mother’s education level	2.289	–1.040	.129	3.070	–1.292	.079
Father’s educational level	2.066	1.008	.148	1.871	0.950	.168
Age	6.757	–0.231	.112	1.398	–0.089	.238
Marital status	0.000	18.798	.999	0.000	18.820	.999
Annual household income	0.031	–0.130	.869	0.080	–0.208	.778

## 4. Discussion

In recent years, infertility patients have been on the rise year by year, with various causes, and both men and women have certain reasons. For example, women may experience endometriosis and blocked fallopian tubes, while men may experience conditions such as asthenozoospermia and oligozoospermia. Under these circumstances, they lost the right to be parents. In order to help these people, ART technology has been developed and continuously developed, enabling some infertile couples to successfully conceive.^[15–17,26]^ According to statistics, the number of babies conceived through ART technology is relatively large, up to 1 million, accounting for about 3% newborns. However, the safety issues of ART technology have received widespread attention, especially the growth, development, and long-term health of its offspring. Some scholars use literature search to collect relevant information in order to understand the impact of ART technology on children’s health. From the literature review results, it can be seen that with the help of IVF technology, pregnant children have a higher risk of congenital malformations compared to normal pregnant children, but the relevant reasons for the risk are not yet clear.^[18]^ Some scholars analyze the application effect and obstacles of in vitro maturation technology in ART technology, and compare this technology with IVF technology. Compared to IVF technology, the efficiency of this technology is relatively low, and efforts can be made to improve its application from the perspective of training clinical doctors.^[19]^ Some scholars analyze the application of ART technology in order to understand its effects. Among them, multiple pregnancies are more likely to cause complications of ART technology. Reducing the number of embryo transfers is beneficial for reducing the probability of complications and improving the safety of ART technology.^[20]^ To further understand the growth and development of IVF under ART technology, this study took preschool IVFs as the research object, analyzed whether there were differences in growth and development between them and naturally conceived infants in the samez age group, in order to deepen the understanding of IVF.

Overall, the growth and development of children in the group S were comparable to those in the Z group. No significant difference (*P* > .05) showed in age-specific height, age-specific weight, Hb level, trace element content, disease incidence at different stages, Gesell developmental scores, WPPSI scores, intellectual development levels in various dimensions such as geometric shapes, DDST screening results, PDI scores, and MDI scores. Compared to the group Z, a smaller number of children in the group S had PDI scores ≤ 69 and MDI scores ≤ 69 (*P* < .05). Additionally, compared to the group Z, group S had a significantly higher number of children in the who were delivered via cesarean section (*P* < .001). In the analysis of influencing factors, birth environment factors such as family background and mother’s education level had no statistically significant impact on the height and weight of children (*P* > .05). However, among the birth environment factors, the father’s education level had a significant impact on PDI, with statistical significance (*P* < .05), and the mother’s level of education had a significant impact on MDI (*P* < .05).

The results of the analysis of 2 different cohorts indicated no significant difference in the intelligence and physical development of children, and they all develop normally. The education level of parents can affect the growth of children. Some scholars conduct research through literature search to understand the impact of ART technology on the production and development of IVF, in order to understand the related growth situation between IVF and naturally conceived infants. With the results of the meta-analysis, no significant difference showed in height between IVF and naturally conceived infants, and there is no statistical significance.^[21]^ Some scholars focus on children and analyze the impact of equal sharing of education among parents. From the analysis results, it can be seen that parents sharing educational responsibilities equally is beneficial for improving children’s cognitive abilities.^[22]^ When a certain scholar conducted research on children’s development, he focused on the role of the father and believed that the involvement of the father and mother in a child’s life can promote their adaptability and other aspects.^[23]^ At present, most studies believe that IVF technology has no significant impact on the mental health of full-term single fetuses, but the relevant studies are few and the sample size is small, and further research is still needed in the future. Some scholars believe that family background such as parents’ education level, social class and maternal age may have a greater impact on offspring’s socio-emotional development and cognitive impairment than the method of fertilization. But parents’ excessive attention and anxiety will reduce their children’s emotional regulation ability,^[24,25]^ There are also some studies that suggest that ART use may be associated with a higher risk of ASD in offspring. However, further prospective, large-scale and high-quality studies are still needed.^[29,30]^

In summary, the growth and development of IVF in the early stage of school age is comparable to that of naturally conceived children at the same time. The high-level of education of parents is beneficial for helping preschool children develop their mental and physical abilities. There are certain shortcomings in the research, and follow-up work on IVF should continue. By increasing the sample size, we can prevent the impact of sample bias on the data.

## Author contributions

**Conceptualization:** Chunyan Guo, Lixin Yang, Jingcai Wang, Qili zhou.

**Data curation:** Qili zhou.

**Funding acquisition:** Chunyan Guo, Qili zhou.

**Investigation:** Chunyan Guo, Lixin Yang, Jingcai Wang, Qili zhou.

**Methodology:** Chunyan Guo, Lixin Yang, Jingcai Wang, Qili zhou.

**Project administration:** Lixin Yang.

**Supervision:** Jingcai Wang, Xia Liu, Yanqiu Wu.

**Writing – original draft:** Chunyan Guo, Lixin Yang, Jingcai Wang, Qili zhou.

**Writing – review & editing:** Chunyan Guo, Lixin Yang, Jingcai Wang, Xia Liu, Yanqiu Wu, Qili zhou.
